# Fluoroquinolones: Neurological Complications and Side Effects in Clinical Practice

**DOI:** 10.7759/cureus.54565

**Published:** 2024-02-20

**Authors:** Ahmed I Anwar, Lei Lu, Connor J Plaisance, Charles P Daniel, Chelsi J Flanagan, Danielle M Wenger, David McGregor, Giustino Varrassi, Adam M Kaye, Shahab Ahmadzadeh, Elyse M Cornett, Sahar Shekoohi, Alan D Kaye

**Affiliations:** 1 Psychology, Quinnipiac University, Hamden, USA; 2 Neurology, Medical University of South Carolina, Charleston, USA; 3 School of Medicine, Louisiana State University Health Sciences Center, Shreveport, USA; 4 School of Osteopathic Medicine, University of the Incarnate Word, San Antonio, USA; 5 School of Medicine, University of Arizona College of Medicine, Phoenix, USA; 6 Anesthesiology, Louisiana State University Health Sciences Center, Shreveport, USA; 7 Pain Medicine, Paolo Procacci Foundation, Rome, ITA; 8 Pharmacy Practice, Thomas J. Long School of Pharmacy and Health Sciences University of the Pacific, Stockton, USA

**Keywords:** neurotoxicity, neurological, fluoroquinolones, neurological system, effects, quinolones

## Abstract

Fluoroquinolones, a popular antibiotic class that inhibits nucleic acid synthesis of bacteria by disrupting the activity of the enzyme’s topoisomerase IV and DNA gyrase, are used to treat bacterial infections. However, the widespread use of these drugs has allowed for the development of microbial resistance in recent years. Quinolones also have many clinically relevant side effects, including psychosis, confusion, seizures, headaches, dizziness, and nausea. Common side effects include tendinitis, myopathy, depression, and fatigue. Cardiovascular side effects include increased risk of aortic aneurysm, aortic dissection, and QT interval prolongation. Overall, quinolones can be an effective choice for treating bacterial infections. Still, the side effect profile and decreased efficacy secondary to microbial resistance no longer make the quinolone class an ideal choice for many types of infection. A better understanding of the role of quinolone-mediated or neurological damage, cardiovascular impairment, and musculoskeletal involvement is imperative to determine the risks/benefits for the clinician.

## Introduction and background

Quinolones, developed in the 1960s, are primarily employed when treating bacterial infections. They function by inhibiting bacterial nucleic acid synthesis and disrupting the activity of enzymes such as topoisomerase IV and deoxyribonucleic acid (DNA) gyrase [1]. Gyrase, an enzyme, functions by unraveling DNA strands, untangling knots within them, and introducing negative supercoils to alleviate topological strain. Topoisomerase, as an enzyme, performs single-stranded cuts and moves across double-stranded DNA [2]. These two enzymes play a crucial role in the replication of bacterial DNA with minimal imperfections and, consequently, serve as the target in the mechanism of action of the quinolone class. Quinolones enter cells through porins, inhibiting DNA replication in bacterial cells and, in turn, hindering bacterial growth and the spread of infection. Due to their effectiveness, quinolones find widespread use in treating infections. These drugs have been subject to misuse and inappropriate prescriptions, which lead to microbial resistance within the drug class [1]. Microbial resistance has significantly reduced the efficacy of the quinolone drug class, making the treatment of susceptible pathogens increasingly challenging. Beyond microbial resistance, other issues are associated with quinolone use, including adverse side effects. The side effect profile of quinolones affects multiple systems in the body, encompassing the neurological, cardiovascular, musculoskeletal, and endocrine systems. This article will delve into the side effect profile of quinolones, their physiologic interactions within the body, and relevant studies in the current literature on this topic. 

## Review

Neurological side effects of quinolones 

Research indicates that quinolones exhibit central and peripheral neurotoxicity [3]. The neurotoxic effects of quinolones encompass antibiotic-associated encephalopathy, seizures, peripheral neuropathy, and exacerbation of myasthenia gravis. It is crucial to comprehensively assess the risks and benefits of prescribing these medications. 

Neurological side effects of fluoroquinolones

Encephalopathy induced by quinolones typically presents with psychosis, often emerging within days of initial treatment [4]. Frequently reported fluoroquinolones associated with encephalopathy include ciprofloxacin, moxifloxacin, levofloxacin, and olfloxacin. In a study involving 631 hospitalized veterans receiving oral and intravenous quinolone treatment for at least 48 hours, 3.7% experienced quinolone-associated psychosis or delirium. The Adverse Drug Reaction Probability Scale, used to assess the causal relationship between drug use and adverse events, yielded a score of 3 in this study [5]. This score suggests a potential association between quinolone use and the development of psychosis or delirium. Notably, elderly patients face higher odds of experiencing neuropsychiatric events [6]. Peripheral neuropathy is linked to system exposure to quinolones as well [7]. In a study by Morales et al., which followed 5357 patients with incident peripheral neuropathy matched to 17285 controls, those taking oral fluoroquinolones had significantly increased risks. The risks rose by 3% for each additional day of current exposure and persisted for up to 180 days after exposure [8]. 

Furthermore, the exacerbation of myasthenia gravis is a severe complication associated with fluoroquinolone use [7]. A retrospective study examining postmarking reports from the United States Food and Drug Association (FDA) and reviewing literature identified 37 unique cases from 1970 to 2011 where exacerbation of myasthenia gravis was suspected to be related to fluoroquinolone exposure. The exacerbation developed a median of one day following exposure. Seizures are a significant concern when using fluoroquinolones, particularly in the presence of renal dysfunction, a history of epilepsy, and brain lesions [9,10]. Although quinolones are thought to lower seizure thresholds, the evidence of an association between antibiotics and symptomatic seizures varies from low to very low, per a 2015 systemic review by Sutter et al. [10]. Another systemic review, encompassing 140 studies involving 21884 children, reached similar conclusions, suggesting a low risk of seizures associated with fluoroquinolone, even for pediatric patients with known brain lesions [11]. 

Fluoroquinolones: ciprofloxacin

Ciprofloxacin, a commonly prescribed fluoroquinolone, is an antibiotic for treating bacterial infections [12]. The widespread use of fluoroquinolones is evident, with nearly 30 million prescriptions written by American doctors in 2016. Despite their everyday use, the FDA has identified various side effects associated with antibiotics, leading to a Boxed Warning, the most severe cautionary label issued by the FDA. This warning highlights side effects such as peripheral neuropathy and central nervous system (CNS) effects. Notably, ciprofloxacin has been demonstrated to negatively impact the quality of life in patients. In a personal account from a 24-year-old individual, ciprofloxacin is described as having a profound and detrimental effect on his life, resulting in the loss of his former self. This patient has received a diagnosis of fluoroquinolone-associated disability (FQAD), a condition linked to the use of fluoroquinolone antibiotics [13]. FQAD affects multiple body systems, including the musculoskeletal and nervous systems. This particular patient was prescribed ciprofloxacin for a UTI and gastritis, and within two weeks, they experienced what is referred to as being "flexed." This term describes fluoroquinolone toxicity, where the body undergoes mitochondrial damage and oxidative stress due to the adverse effects of fluoroquinolone antibiotics. 

FQAD

FQAD arises following fluoroquinolone antibiotics, commonly prescribed for antimicrobial treatments [14]. Potential therapies for FQAD include strategies to reduce oxidative stress, restore mitochondrial potential, and employ other mitochondrial-related therapies. Fluoroquinolones impede bacterial deoxyribonucleic acid (DNA) unwinding and duplication, aiding in the treatment of bacterial infections. However, the FDA has identified numerous side effects associated with fluoroquinolone use, with one notable impact being the disruption of the nervous system. Symptoms such as insomnia, restlessness, seizures, and convulsions have been reported in connection with fluoroquinolone treatment. 

Fluoroquinolone-induced neurotoxicity

Quinolones are linked to various neurotoxic effects including confusion, and psychosis [15]. Ciprofloxacin, a specific type of quinolone, is associated with altered mental status and confusion in patients. Olfloxacin, which has higher permeability in the CNS, has fewer reported cases of neurotoxicity. Levofloxacin has been reported to induce delirium with psychotic features and seizures [16]. Quinolones also have been linked to dyskinesias and Tourette-like syndromes [17]. Encephalopathy is another expected manifestation of neurotoxicity. Fluoroquinolone-induced CNS toxicity involves the inhibition of GABA receptors and the activation of N-methyl-D-aspartate (NMDA) receptors [15]. Studies in rats suggest that compromised blood-brain barriers may elevate drug concentrations and increase neurotoxic effects. The disruption of the GABAergic system is also implicated in fluoroquinolone-induced seizures. 

Quinolone-induced painful peripheral neuropathy 

A case report presented by Estofan et al. involved a 20-year-old patient with type 1 diabetes who experienced severe painful peripheral neuropathy during a 10-day course of levofloxacin, a quinolone, for epididymitis [18]. The pain reached a score of 10/10, which led to the transfer to an inpatient pain unit where aggressive treatment had shown minimal improvement. A skin biopsy confirmed small fiber neuropathy, and subsequent treatment with intravenous immunoglobulin provided relief. Diabetes puts patients at higher risk for peripheral neuropathy, and fluoroquinolone use has been shown to increase this risk. Data has shown a link between fluoroquinolones and adverse outcomes in the peripheral nervous system. The patient's side effects emphasize the caution needed when prescribing fluoroquinolones. 

Mechanism of neurotoxicity of quinolones

Though it is not fully understood, several proposed mechanisms of quinolone-associated neurotoxicity exist. First, quinolones pass through the blood-brain barrier and affect excitatory and inhibitory neurological pathways. Based on an in vitro model of the hippocampus slice model, the NMDA receptor is a possible target of fluoroquinolones [19]. The commonly used compounds range from least to most excitatory: ofloxacin, ciprofloxacin, nalidixic acid, moxifloxacin, fleroxacin, lomefloxacin, enoxacin, clinafloxacin, tosufloxacin, and trovafloxacin [20]. Secondly, the concomitant use of quinolones with other medications can exacerbate this neurotoxicity - quinolones have various interactions with other medications [19]. Lastly, the toxicity of fluoroquinolones will be exacerbated in the context of pre-existing conditions, such as acute or chronic renal failure, hepatic failure, systemic infections, or prior history of neurological pathologies [10].

Effects of quinolones on the cardiovascular system

Quinolones have demonstrated adverse effects on multiple systems in the body, including the cardiovascular system. In 2019, the FDA warned about the correlation between fluoroquinolone use and the development of aortic dissection [21]. The FDA advises clinicians not to prescribe fluoroquinolones for patients with an aortic aneurysm or patients at risk for an aortic aneurysm [22]. This is a rare but severe, life-threatening event, and its possibility should be considered when choosing antibiotic therapy for patients. As such, those with pre-existing heart conditions and people of older age are not recommended to take fluoroquinolones due to the increased risk of this cardiovascular event. A study was conducted by Rawla et al. in 2019 to evaluate the risk of developing either aortic aneurysm or dissection following fluoroquinolone administration. It was noted that fluoroquinolone administration doubled the risk of developing an aortic aneurysm within sixty days of the initial exposure. Of note, the risk was higher in females than males and more elevated in older patients [23]. Quinolones have also been found to cause QT interval prolongation by blocking voltage-gated potassium channels. Because of this effect, quinolones should be used cautiously or avoided entirely in patients with torsades de pointes (TdP) history. This polymorphic ventricular tachycardia requires immediate intervention. The risk of TdP with fluoroquinolone use is low. However, its occurrence is possible and should be considered, especially when prescribing fluoroquinolones with other QT-prolonging medications [24].

Effects of quinolones on the musculoskeletal system

Though widely used clinically as a standard treatment due to their potent antimicrobial ability, (*Quinolones)* quinolone has been shown to cause adverse effects in the musculoskeletal system specifically tendons, ligaments, and cartilage. These effects have been studied at both intracellular and extracellular levels. Many studies have described these effects on the musculoskeletal system, including altering the extracellular matrix structures and the induction of apoptosis [25-27]. Multiple case reports show evidence of increased tendinopathy risk in patients taking fluoroquinolones concomitantly with glucocorticoids [28]. Inside the extracellular matrix, quinolones have been shown to enhance the expression of matrix metalloproteinases (MMPs), leading to degradation [25,29,30]. Studies have also provided evidence of the synergistic effects of fluoroquinolone and steroid treatments on ligaments. Studies provide evidence that steroids and fluoroquinolones exhibit cytotoxic effects on ligamentous fibroblasts, with fluoroquinolones (such as ciprofloxacin) inducing apoptosis and steroids inducing cell senescence. However, under both treatments, there was reduced cell viability and decreased collagen type I production [27]. This was shown in a study byLuciani et al. on the effects of fluoroquinolones and steroids on human ligament cells, where ciprofloxacin and methylprednisolone were administered to human ligament cells. After 7 and 14 days of culture, they were evaluated for cell viability, senescence-associated β-galactosidase staining, and collagen type I expression [26]. These effects were found in a similar study conducted by Sendzik et al., where human-derived tenocytes were incubated with dexamethasone and ciprofloxacin or levofloxacin. Tenocytes are cells embedded in tendons and offer tensile strength by producing collagen fibers and elastic fibrils [31]. When treated with either ciprofloxacin or levofloxacin at three mg/L and ten mg/L, respectively, they were shown to increase caspase 3, an apoptotic cell marker. Sendzik et al. also showed an alteration in the expression of β1-integrin, a crucial signal transduction receptor in the mitogen-activated protein kinase (MAP) pathway. The effects that quinolones were discovered to have on this specific pathway induced apoptosis at levels that are seen clinically when patients are prescribed this medication. When these cells were treated with 0.1 μM and ten μM of dexamethasone, the number of MMPs and activated caspase three proteins was increased, and these increases were exacerbated by introducing quinolone to the tenocyte culture. This demonstrated that the cytotoxic effects of steroids and quinolones are significantly increased when taken synergistically [32]. While there is much literature on the effects of quinolones on connective tissue, the exact mechanism by which these effects occur is still widely unknown.

Effects of quinolones on glucose

Though rare, fluoroquinolones have been shown in numerous case studies to induce hypoglycemia in patients while taking drugs such as levofloxacin and ciprofloxacin [23,33]. Currently, there is not much-published literature detailing the specific correlation mechanism between hypoglycemia and quinolones. However, many published cases support the theory that it is possible to develop hypoglycemia while taking these medications. In a randomized controlled trial composed of 48 men and women diagnosed with non-insulin-dependent type 2 diabetes, participants were given either gatifloxacin, ciprofloxacin, or a placebo to test the effects of these quinolones on glucose metabolism and insulin levels. Serum glucose and insulin levels were measured daily, and an increase in insulin was seen in the group treated with 400 mg of gatifloxacin. However, it was a transient increase only lasting for a short period and was considered not statistically significant long-term nor clinically substantial [25,32]. Wendy S. Biggs reported multiple cases where patients were admitted to the hospital for different reasons and experienced hypoglycemia after beginning treatment with gatifloxacin. It is noted that not all the patients in these cases had a diagnosis of diabetes. However, all patients took gatifloxacin with glyburide, a glucose level-controlling medication. The symptoms induced by the hypoglycemia were not resolved until the gatifloxacin treatment was discontinued [25]. Theodoros Kelesidis and Elvia Canseco reported the case of a 65-year-old woman with type 2 diabetes, chronic kidney disease, and liver cirrhosis who was prescribed oral ciprofloxacin to treat a urinary tract infection [34]. The patient had a history of hypoglycemia-related to treatment with levofloxacin two months prior. It was noted she was also taking glipizide to control her diabetes and glucose levels; however, the final dose of glipizide was taken six hours before initiating treatment with ciprofloxacin. She experienced seizures and severe hypoglycemia (20 mg/dL). Serum studies found an increase in insulin and increased C-peptide levels, which suggested she was experiencing hyperinsulinemic hypoglycemia. This patient’s glucose levels were stabilized after treatment with octreotide [26]. A case involving a 66-year-old male with uncontrolled diabetes was reported to have hypoglycemia after receiving treatment for a UTI with moxifloxacin. The patient was not receiving any treatments for the control of his diabetes or serum glucose levels. The hypoglycemia resolved in this patient once moxifloxacin was discontinued [27].

Mechanism of quinolone-induced hypoglycemia

As stated previously, the exact mechanism of hypoglycemia in patients treated with fluoroquinolones is unknown. However, it has been postulated that a possible mechanism could be due to blockage of the adenosine 5’-triphosphate-sensitive potassium channels in pancreatic β-cells that are responsible for regulating calcium influx and subsequently augment insulin release [27,29].

Prescription of quinolones

There have been needless and unapproved prescriptions of quinolones for infections that could be treated with safer first-line medications. In November 2015, the FDA advisory committee focused on the side effects that lead to potentially irreversible impairment with quinolone usage [35]. The FDA committee concluded that serious risks outweighed the benefits for infections such as bacterial sinusitis, bronchitis, and urinary tract infections, especially when alternative treatments were available. A drug safety communication in May of 2016 recommended reserving fluoroquinolones for conditions only when there were no other options that existed due to the possibility of permanent, disabling side effects that can occur. The new Boxed Warning also contains recent limitations-of-use statements that reserve fluoroquinolones for patients with no other treatment options for these diseases.

Fluoroquinolone resistance 

Increased prescription of fluoroquinolones has led to increased resistance [36]. The cause of resistance to fluoroquinolones arises due to alterations in the enzymes DNA gyrase and topoisomerase IV. Mutations result in changes to these enzymes, which cause further resistance. The resistance to quinolones is also mediated by plasmids producing the Qnr protein. The Qnr portent protects the quinolone targets from inhibition, increasing the drug's resistance. 

Discussion

The neurological side effects associated with quinolone use include encephalopathy [4], peripheral neuropathy [8,31], seizures [10], and exacerbation of myasthenia gravis [7] due to broad polypharmacy interactions of quinolones when using other drugs [10], systemic quinolone exposure, and quinolones’ ability to cross the blood-brain barrier and target the NMDA receptor [18]. Studies assessing the cardiovascular side effects of quinolone use indicate an increased risk of aortic aneurysm [21], aortic dissection [18], and QT interval prolongation due to mechanistic blocking of voltage-gated potassium channels [16]. The broad range of musculoskeletal side effects, including tendinopathy [30], decreased type I collagen production [29], and both ligament and cartilage damage associated with quinolone use, is indicated due to synergistic cytotoxicity with glucocorticoid use and altered expression of apoptosis-inducing factors, including MMPs, β1-integrin, and caspase 3 [31]. Lastly, the literature suggests quinolone use has been associated with hypoglycemia [32]. However, this reaction is rare, and the mechanism is still unclear with suggested pancreatic potassium channel interaction [29,37]. With increasing bacteria resistance and evidence suggesting severe and even life-threatening side effects due to quinolone use, one must consider the predisposing risks [38], face a lack of alternative antibiotic treatment [39], and practice close surveillance during patient use when considering quinolone use in antibiotic therapy. Quinolones have been seen to have multiple side effects that can debilitate patients (Table 1). Caution when prescribing quinolone should be taken, and exploration of other treatment options should be exhausted before using quinolones. 

**Table 1:** Reviews of Quinolone Safety and Adverse Drug Reactions

Author (Year)	Groups Studied and Interventions	Results and Findings	Conclusions
Scavone et al. (2020)[39]	The study reviewed Individual Case Safety Reports (ICSRs) and online public reporting systems (RAM systems) from 2001-2019 associated with quinolone use to assess the most prevalent risk factors for musculoskeletal, neurological, and psychiatric adverse events.	The study identified 87 case reports involving at least one adverse neurological, musculoskeletal, and psychiatric event. The risk factor with the highest link to all three adverse effects was “age greater than 60 years”, reported in 69.5% of the cases. Other reported risk factors included “therapeutic indication” and “renal failure”.	Patients over age 60 should be especially aware of their predisposed risk to adverse reactions associated with quinolone use.
Huruba et al. (2022) [33]	The study reviewed case reports from Vigibase, which described fluoroquinolone-induced adverse drug reactions involving the peripheral nervous system.	The study identified 6331 adverse drug reactions involving the peripheral nervous system reported amongst at least three different fluoroquinolone drugs. The most common side effect was peripheral neuropathy (5492 reports).	This case review details safety concerns regarding fluoroquinolone use and the possibility of potent adverse drug reactions in the peripheral nervous system.
Mathews et al. (2019)[40]	The retrospective cohort study investigated 482 patients prescribed fluoroquinolones compared to 318 patients prescribed other antibiotics for seven months to determine the incidence and predisposing risk factors for adverse drug reactions with fluoroquinolones.	The study found that 8.5% of patients taking fluoroquinolones developed adverse drug reactions, while only 4.1% of patients on other antibiotics developed adverse drug reactions. Levofloxacin had the highest incidence of adverse drug reactions of all the drugs reviewed. Fluoroquinolone use should therefore be limited to conditions where alternatives are unavailable.	The data suggests an increased incidence of adverse drug reactions and decreased safety with fluoroquinolone use.

## Conclusions

Quinolones have demonstrated pharmaceutical use as antibiotics for various antimicrobial infections; however, current literature suggests many adverse indications and side effects, including neurological impairment, cardiovascular impairment, musculoskeletal involvement, and hypoglycemia. Due to their widespread use as antibacterial agents, there is an increasing challenge of antibiotic resistance to quinolones. A great deal of data has been made available regarding significant risks patients may face when using this class of drugs.
